# Polycystic Ovary Syndrome: Effect and Mechanisms of Acupuncture for Ovulation Induction

**DOI:** 10.1155/2013/762615

**Published:** 2013-09-02

**Authors:** Julia Johansson, Elisabet Stener-Victorin

**Affiliations:** ^1^Institute of Neuroscience and Physiology, Department of Physiology, Sahlgrenska Academy, University of Gothenburg, Box 434, 405 30 Gothenburg, Sweden; ^2^Department of Obstetrics and Gynecology, First Affiliated Hospital, Heilongjiang University of Chinese Medicine, Harbin 150040, China

## Abstract

Polycystic ovary syndrome (PCOS), the most common endocrine disorder among women of reproductive age, is characterized by the coexistence of hyperandrogenism, ovulatory dysfunction, and polycystic ovaries (PCO). PCOS also represents the largest part of female oligoovulatory infertility, and the management of ovulatory and menstrual dysfunction, comprises a third of the high costs of PCOS treatment. Current pharmacological and surgical treatments for reproductive symptoms are effective, however, associated with negative side effects, such as cardiovascular complications and multiple pregnancies. For menstrual irregularities and ovulation induction in women with PCOS, acupuncture has indicated beneficial effects. This review will focus on the results from randomized controlled acupuncture trials for regulation of menstrual dysfunction and for inducing ovulation in women with PCOS although there are uncontrolled trials with nonetheless interesting results. Animal experimental studies will be further discussed when they can provide a more mechanistic explanatory view.

## 1. Introduction

Although polycystic ovary syndrome (PCOS) has been recognized for more than 70 years there is no cohesive definition, and the diagnosis still causes debate. The most recent and used definitions are the National Institute of Health (NIH), Rotterdam, and Androgen excess and PCOS society (AES) criteria, where all are presently used in clinic (Table 1). In consequence, prevalence is difficult to conclude and depends on the used definition, as well as the ethnicity of the measured population. Most studies report prevalence between 6–15% but sometimes even up to 20%, depending on used criteria [1–3]. The different definitions also result in several PCOS phenotypes with a wide range of severity of the syndrome (Table 1), which comprises concerns when comparing clinical studies using different definitions. A recent NIH workshop on PCOS recommended to explicitly report PCOS phenotypes in all future clinical studies [4, 5]. 

## 2. Pathophysiology and Etiology of PCOS

**Figure 1:**
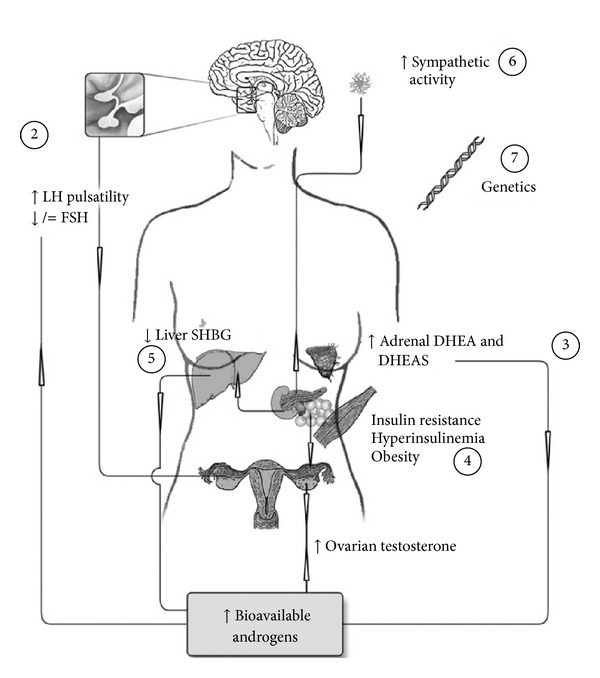
Summary of the PCOS pathophysiology. (1) Ovarian androgens are the main source of hyperandrogenemia in PCOS. Hyperandrogenemia has both a direct effect on the ovarian alterations and (2) an increasing effect on pituitary LH pulse frequency and amplitude with relative low FSH secretion. (3) Further, adrenal androgens contribute to PCOS androgen excess. (4) Insulin resistance with compensatory hyperinsulinemia enhances ovarian androgen production as well as (5) decreases production of SHBG in the liver, and both increase the pool of bioavailable androgens. (6) PCOS is also associated with increased muscle sympathetic nerve activity that is related to high testosterone, insulin resistance, and obesity. (7) Genetic defects probably contribute to the pathology of PCOS. LH: luteinizing hormone, FSH: follicle stimulating hormone, SHBG: sex hormone binding globulin, DHEA: dehydroepiandrosterone, and DHEAS: dehydroepiandrosterone sulphate.

Despite high incidence, the etiology of PCOS remains unknown. Due to the heterogeneity in the representation of clinical and biochemical features, it has been debated whether PCOS actually represents one single disorder or several ones. Symptoms of PCOS often manifest around puberty, but the origin may be programmed already as early as during fetal development [6–8]. One of the most common features of PCOS is insulin resistance, represented in 85% of PCOS women [9]. The other common feature of PCOS, elevated androgen levels, affects around 60–80% of PCOS women and can result in the clinical signs: hirsutism, acne, and, to some extent, alopecia [10]. In PCOS, high circulating levels of androgens, estrogens, sex steroid precursors, and glucuronidated androgen metabolites have been demonstrated by gas chromatography tandem mass spectrometry (GC-MS/MS) and liquid chromatography tandem mass spectrometry (LC-MS/MS) [11]. The major androgen excess in PCOS originates from the ovaries, but the adrenals also contribute to some part [12]. Hyperinsulinemia, often found in PCOS, inhibits the production of sex hormone binding globulin (SHBG), thereby further contributing to levels of free circulating androgens [13].  Figure 1 summarizes the PCOS pathophysiology described below.

Androgens possess a central position in PCOS, are closely related to the ovarian morphology, and are sufficient to cause PCOS-like states in both animal models and female-to-male transsexuals [14, 15]. Both androgens and insulin have therefore been presented as a key underlying cause of PCOS. Since it is not known when or where the pathology actually begins, several different hypotheses are presented. Prenatal androgenization represents an established hypothesis of PCOS etiology and is based on animal models including monkeys, sheep, and rodents where prenatal androgenization inflicts several features of PCOS in the offspring [16–19]. However, in humans, although increased levels of androgens have been found in pregnant PCOS women [20], only one study has found increased levels of testosterone in umbilical vein blood in infants of PCOS mothers although these levels were measured by immunoassays and not mass spectrometry [21]. Prepubertal exposure to androgens is yet another hypothesis that originates in the pubertal start of symptom manifestation [7, 8], from which several animal models of PCOS have been developed [22–24].

Although it is not a part of the diagnosis, PCOS is also strongly associated with insulin resistance and compensatory hyperinsulinemia, type 2 diabetes, and dyslipidemia [25, 26] and the prevalence of women with PCOS being either overweight or obese is high [27]. Both androgens and insulin, which both increase during puberty, are therefore considered to be two key players in PCOS. Most likely both contribute, but it is still unclear which one of these that is most related to the etiology [7, 8].

Asides from these genetics, altered ovarian steroidogenesis [28, 29], and increased sympathetic activity [30] has been proposed as participating causative mechanisms. 

### 2.1. Neuroendocrine Dysfunction in PCOS

In PCOS the disturbed hypothalamic-pituitary-ovarian (HPO) axis has been extensively reviewed [31–34]. The most evident neuroendocrine feature regulating abnormal ovarian follicle development in PCOS is increased luteinizing hormone (LH) pulsatility regarding both frequency and amplitude, with relatively low FSH secretion [35–39]. Increased LH pulse frequency increases theca cell production of androgens, while the lower FSH levels impairs follicle maturation and consequently ovulation [32].

The cause of LH hypersecretion in PCOS is probably due to enhanced pituitary sensitivity to gonadotropin releasing hormone (GnRH) or to changes in GnRH secretion patterns rather than increased GnRH secretion [35, 39, 40]. It appears to be a result of an acquired impaired sensitivity of the hypothalamic pulse generator to the negative feedback of estrogen and progesterone in PCOS, possibly by chronic estrogen exposure [35, 38, 39, 41]. Levels of follicle stimulating hormone (FSH) in PCOS appear to be low or within the lower follicular range, and response to GnRH is relatively similar to ovulatory controls [35]. Altered sex steroid production, metabolic dysfunction, and obesity may all contribute to the changes in LH secretion pattern. Hyperandrogenemia itself may cause hypothalamic desensitization to progesterone/estrogen negative feedback that further increase gonadotropin secretion and hence ovarian androgen production, causing a self-driven viscous circle [42, 43]. Although increased body mass index (BMI) has a blunting effect on LH secretion [36, 39], hyperinsulinemia and insulin resistance may directly or indirectly (by enhancing ovarian gonadotropin stimulated sex steroid production) contribute to the abnormal gonadotropin secretion, although the mechanism is not clear [44–46]. All these factors increase free androgen levels and contribute to anovulation.

### 2.2. Ovarian Dysfunction in PCOS

The ovulatory dysfunction in PCOS can be ascribed to disturbed follicular development with excessive early follicular growth and abnormal later stages of arrested follicle growth well before expected maturation [47]. This pattern of follicular growth with failure in the selection of a dominant follicle for ovulation results in one of the hallmarks of PCOS, PCO morphology. The ovarian dysfunction of PCOS involves both these morphological features of polycystic ovaries, described as an accumulation of small antral follicles of size 2–9 mm, as well as the clinical consequence of oligo-/anovulation. The prevalence of menstrual irregularities oligo-/amenorrhea in PCOS depends on the used diagnostic criteria but is approximately 75% [10]. If we use the NIH criteria, of course all patients will experience menstrual irregularities. Ultimately, irregular ovulation can cause infertility due to difficulties conceiving and should be acknowledged. 

**Figure 2:**
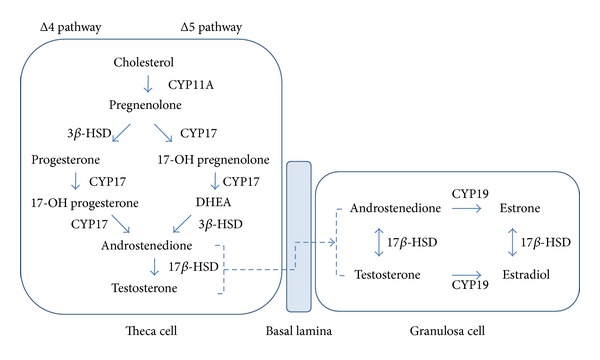
Schematic illustration of steroid synthesis in the ovary.

Moreover, there are several other follicle abnormalities where the most consistent feature is androgen hypersecretion [48]. Ovarian steroid production is based on a close collaboration between theca and granulosa cells in the growing follicles and requires gonadotropin input (Figure 2) [49]. Theca cells produce androstenedione from cholesterol, either by the Δ4 or Δ5 pathway, and the conversion to estrone and estradiol thereafter is exclusively acknowledged aromatase cytochrome P450 hydroxylase (CYP19) containing granulosa cells [50]. Women with PCOS appear to have theca interna hyperplasia, a thicker layer of the theca cells, which seem to be responsible for their increased androgen steroidogenesis. Moreover, each theca cell has increased expression of LH receptors, with increased susceptibility to LH stimulation [33, 50, 51]. 

There are local selectors for follicle recruitment and growth within the ovary that might contribute to the impaired follicle development in PCOS. AMH is expressed by early antral and preantral follicles, but not in later stages of development, and reflects the size and activity of the follicular pool [52, 53]. There is also evidence of its involvement in the regulation of recruitment of primordial follicles into the growing pool, presumably by decreasing the granulosa cell sensitivity to FSH [54]. Adding antimüllerian hormone (AMH) to ovarian cultures reduces the number of growing follicles, while removing the gene increased the proportion [55, 56]. In the small primordial and transitional follicles of anovulatory PCOS, AMH protein expression is reported to be reduced [57]. This may contribute to the inappropriate recruitment of growing follicles. Additionally in both circulation and antral follicular fluid of PCOS women, AMH levels are increased, and these are associated with poor reproductive responsiveness to treatment [58–63]. These high circulating levels may be a reflection of the increased pool of granulosa cells instead of an increased expression. Since high levels of AMH are associated with lower levels of FSH and estradiol, it has been suggested that the AMH excess is involved in the lack of FSH-induced aromatase activity that is characteristic of follicular arrest in PCOS [62, 64]. In addition, testosterone exposure downregulates AMH expression in granulosa cells of small bovine follicles in culture and could possibly represent a mechanistic origin of PCOS [65].

Ovarian inhibins are expressed in ovaries and act as modulators to suppress FSH levels. As a response to increased FSH levels inhibin B is increasingly expressed during early follicular phase in small developing follicles, while inhibin A is selectively produced in the dominant follicle. Inhibin B therefore correlates to total follicle number and may be a marker of follicle quality [66]. But inhibins also have a local effect to stimulate androgen production synthesis in theca cells for estradiol production [66–68]. Most PCOS studies find no differences in basal circulating inhibin B, but an abnormal and increased response to FSH. The normal basal levels may be explained by the diminished FSH secretion in PCOS or that the follicle quality is lower. The increased response to FSH could simply be due to their increased number of preantral and small antral follicles [67–70]. Moreover, follicular arrest in PCOS is associated with reduced levels of both inhibin A and inhibin B in follicular fluid, which both could explain the normal circulating levels although their increased pool of growing follicles, but also making these to possible actors in the impaired follicle development [71]. There is low evidence of any diagnostic value of circulating basal inhibin B measurements in PCOS [67, 72].

Taken together, impaired folliculogenesis and steroidogenesis in PCOS seem to be multifactorial and are probably influenced by extra ovarian factors such as androgens, insulin, neuroendocrine alterations, and intraovarian local and intrinsic factors.

### 2.3. Increased Sympathetic Activity

The autonomic nervous system consists of two divisions: the sympathetic and the parasympathetic nervous systems, and is controlled by the neurotransmitters noradrenaline and adrenaline and activation of adrenergic receptors. In a normal, healthy state, a fine balance between these two divisions, ensures homeostasis. Many of the classical components of PCOS such as polycystic ovaries, insulin resistance with related hyperinsulinemia, central obesity, and hypertension are associated with increased sympathetic activity [73–76]. It has therefore been suggested to account for at least a part of the syndrome etiology [74, 75, 77]. That increased sympathetic innervation of the ovaries might contribute to the impaired follicular development in PCOS that is supported by clinical evidence such as increased density catecholaminergic nerve fibers, increased NGF production, and altered catecholamine metabolism and/or uptake in PCOS ovaries [75, 78, 79]. Heart-rate recovery after a bout of exercise and heart-rate variability can be used as noninvasive markers of autonomic function. Measures in PCOS women indicate that they have decreased dynamic activity in their autonomic function, possibly by decreased activity in the parasympathetic component and increased in the sympathetic component [80–83]. These are though indirect measures and their accuracy may be questionable. We have though demonstrated by microneurography, which is a direct and reliable measure of muscle sympathetic nerve activity (MSNA), that women with PCOS have an increased sympathetic nerve activity that is correlated to high levels of testosterone [30].

## 3. PCOS: A Well-Orchestrated Pathology

Androgens play a central part in the pathology of PCOS. Androgens alone can affect many of the systems that are impaired in the syndrome and are sufficient to cause PCOS-like states in both animal models and female-to-male transsexuals [14, 15, 17–19, 84–86]. But these alterations can themselves further increase hyperandrogenemia. Consequently, a vicious circle is created where the individual pieces may augment each other, although it is not clear where it started. Hyperandrogenemia in PCOS originates mainly from the ovaries and can have central effects by increasing gonadotropin secretion via affecting sex-steroid feed-back systems as well as enhancing the effect on ovarian gonadotropin stimulated sex steroid production [12, 42, 43]. Androgens also directly impair follicle development and maturation and thereby contribute both to the PCO morphology and to the ovarian pool of androgen producing cells [12]. Both of these will further drive ovarian androgen production and increase levels of free circulating androgens. Additionally, although the mechanism is not completely clear, increased adrenal androgen production contributes to the androgen excess in PCOS [87, 88].

Androgens are also associated with an atherogenic blood lipid profile, enlarged adipocyte size, and peripheral insulin resistance although the androgen excess may not be the primary cause of their insulin resistance [89–91]. Moreover, together with obesity, this increases the risk of type 2 diabetes and cardiovascular disease (CVD) [27]. Similar to androgens, insulin resistance and hyperinsulinemia enhance ovarian gonadotropin stimulated sex steroid production [12, 44–46] and may contribute to the abnormal gonadotropin secretion although the mechanism is not clear [44–46]. Hyperinsulinemia also decreases liver production of sex hormone binding globuline (SHBG) which increases the amount of bioavailable free circulating sex steroids [13]. 

PCOS is related to increased MSNA and of special interest is that testosterone concentration was found to be a strong independent predictor [30]. Increased sympathetic nerve activity is related to insulin resistance with related hyperinsulinemia, central obesity, and hypertension [73–76] and might contribute to the increased cardiovascular risk [30]. There is also evidence that supports an increased sympathetic nerve activity to the ovaries [75, 78, 79, 92] that may further drive androgen production and PCO morphology [93]. Apart from the previously described factors, there is a strong genetic component with familial aggregation of cases and symptoms [94, 95] that probably is involved in the etiology of PCOS. Altogether, PCOS is a coordinated pathology containing factors that strongly influence each other in a viscous circle, making it difficult to separate the etiology.

## 4. Hypothetical Mechanism of Acupuncture

Acupuncture is widely practiced and is now also accepted in the western world for the treatment or adjunct treatment for more and more conditions [96]. It is a relatively safe treatment with few side effects [97].

**Figure 3:**
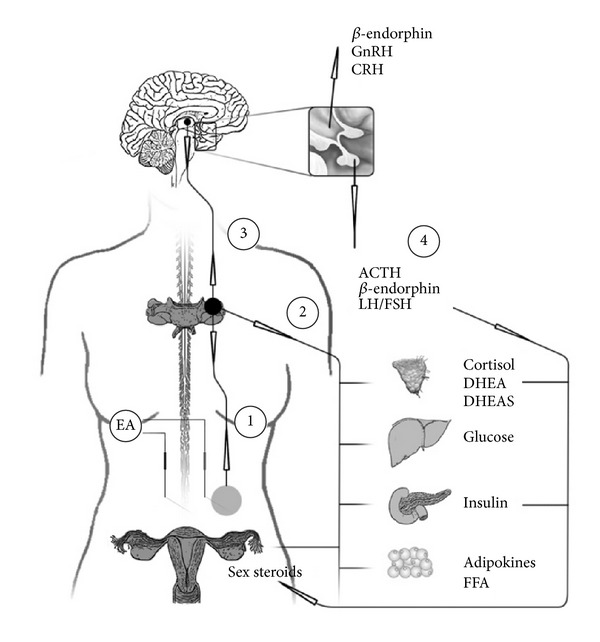
Schematic illustration of a hypothetical mechanism explaining the effects of acupuncture in PCOS. (1) Stimulation of acupuncture needles in skeletal muscle excites ergoreceptors that activate afferent sensory nerve fibers. These signals are transmitted to the spinal cord where they (2) through spinal reflexes may modulate the sympathetic output to the target organs in the same area of innervation. (3) Signals also reach the central nervous system via supraspinal pathways where they can exert central effects. Hypothalamic *β*-endorphin is implicated in the effect of acupuncture. It modulates the autonomic system but may also alter the release of GnRH and CRH. (4) These can enable an effect on reproductive function (via LH and FSH), adrenal function (ACTH), and pancreatic function (circulating *β*-endorphins). (ACTH: adrenocorticotrophic hormone, CNS: central nervous system, CRH: corticotrophin releasing hormone, EA: electroacupuncture, FFA: free fatty acids, FSH: follicle stimulating hormone, GnRH: gonadotropin releasing hormone, and LH: luteinizing hormone).

Acupuncture originates from traditional Chinese medicine (TCM) where fine needles are placed in the skin and underly muscle tissue at specific areas of the body, so-called acu- or acupuncture points. When placed, needles are stimulated manually by rotating or perturbing needles up and down, so-called manual acupuncture. Needles may also be stimulated electrically by applying an electrical field and passing an electrical current between two needles, so-called electroacupuncture (EA). Low-frequency (1–15 Hz) electroacupuncture with an intensity that evokes muscle contractions is believed to achieve biological processes that resemble the effects of exercise. Acupuncture from a western scientific perspective cannot confirm point specificity and instead explain the effect with the activation of afferent sensory nerve fibers at appropriate segmental level [98, 99]. Acupuncture activates and modulates nervous pathways at peripheral (local), segmental (in the spinal cord), and supraspinal levels within central nervous system (CNS).  Figure 3 illustrates the hypothetical mechanism explaining the effects of acupuncture in PCOS.

Starting at a peripheral level, both manual and electrical stimulations increase glucose uptake and microcirculation [100–103]. When needles are inserted and stimulated, peripheral nerve terminals release several neuropeptides, such as neuropeptide Y (NPY), vasoactive intestinal polypeptide (VIP), substance P, and calcitonin gene-related peptide (CGRP), and give an immediate local reaction, by which the two latter are probably involved, with an increase in microcirculation [100, 104, 105]. Moreover, data from administration of the opioid antagonist naloxone also suggest low-frequency EA stimulated peripheral opioid release [106].

Mechanoreceptors, responding to mechanic pressure or distortion, are activated by muscle contractions and are suggested to be involved in the somatic response of both manual and electrical stimulations [107, 108]. Activation of mechanoreceptors by manual or electrical stimulation activates sensory nerve fibers, myelinated A*α*, *β*, *δ*, and unmyelinated C-fibers [109, 110]. These signals are transmitted to the spinal cord (segmental level) where they through spinal reflexes may modulate the sympathetic output to the target organs in the same area of innervation as where needles are placed [111]. Importantly, these signals are also controlled via supraspinal pathways within the central nervous system (CNS) [112]. 

The efficacy of acupuncture for analgesia has been extensively studied and widely used. The involvement of endogenous opioids within the central nervous system has been suggested to mediate the effect of acupuncture-induced analgesia and lowering of blood pressure [113–116]. There are three opioid receptors *μ*, *δ*, and *κ* found in peripheral afferent nerve terminals and within the central nervous system in areas related to nociception and pain [117, 118]. *β*-endorphin, one of the cleavage products of proopiomelanocortin (POMC), binds to the *μ*-receptor with high affinity and has received special attention [117]. *β*-endorphin produced in the arcuate nucleus of the medial basal hypothalamus is released within CNS, but it is also produced in the pituitary where it is released into the peripheral circulation [119–121]. The *β*-endorphin is produced in hypothalamus project to the midbrain (especially the periaqueductal grey) and brainstem nuclei where it can influence pain sensitivity and autonomic function [120, 121]. The effect on autonomic function includes effects on the vasomotor centre with regulation of blood pressure and muscle sympathetic nerve activity (markers of sympathetic tone) [122]. The other system is also under hypothalamic control, by corticotrophin releasing hormone (CRH), and involves the anterior pituitary lobe where POMC is cleaved into equal amounts of *β*-endorphin, melanocyte stimulating hormone, and adrenocorticotrophic hormone (ACTH) that is released into circulation [123]. The two systems work independently, but both can be activated by afferent nerve activity such as manual and low-frequency electrical stimulation of acupuncture needles and exercise [120]. 

The *β*-endorphinergic system is involved in many physiological effects, both centrally and peripherally, such as reproductive function, analgesia, stress response, and carbohydrate metabolism [124]. Both circulating and central levels of *β*-endorphin have been shown to be modulated by acupuncture [125–127]. Further implications of acupuncture involving modulation of autonomic function are the reducing effect on MSNA and blood pressure [128, 129]. The pain reliving effect of acupuncture was further blocked by low doses of naloxone, an opioid receptor antagonist, and the blood pressure suppression by high doses of naloxone [129, 130].

The connection between central *β*-endorphin and reproductive function involves both direct and indirect tonic inhibitory effects on GnRH and subsequent LH release and possibly also GnRH biosynthesis [121, 131]. It is generally accepted that opioids mediate the inhibitory effect of estrogen [132]. Moreover, a reduction in opioid inhibitory tone amplifies and is essential, for generation of the LH surge preceding ovulation [121, 133].

Circulating *β*-endorphin is considered to be more related to stress stimuli than actually reflecting central opioid activity and should not be used as a marker of central activity [134]. Still, the changes in plasma *β*-endorphin provide a link to the hypothalamus pituitary (HPA) axis, since *β*-endorphin is co-released with ACTH from the pituitary [123]. The controlling factor CRH is released by stress, but it is also known to decrease GnRH secretion, linking it back to the reproductive axis [135]. Acupuncture has been shown to decrease levels of CRH within hypothalamus and may therefore present effects on both HPO and HPA axes [136].

### 4.1. Control Situations of Acupuncture?

In general the effect of acupuncture may also be influenced by the patient's own expectations, since there is a strong psychological component to it [137]. The use of placebo in acupuncture experiments is therefore always under debate, and efforts to develop suitable control situations have been made. However, the use of so-called placebo needles or minimal/sham acupuncture may not be a true control since clinical studies suggest that they are not inert treatments [138, 139]. 

### 4.2. Implications of Acupuncture for Ovulation Induction in PCOS

Since many of the features of PCOS are associated with disturbed opioid or sympathetic tone, including disturbed gonadotropin secretion, insulin resistance, and central obesity [74, 124], opioid and/or sympathetic tone may be implicated in the pathogenesis of the condition. Women with PCOS have higher levels of circulating *β*-endorphin which may contribute to the pathogenesis, possibly by insufficient inhibition of GnRH by *β*-endorphins in the CNS [124, 140–142]. This is supported by studies in which the *μ*-receptor antagonist naltrexone improves cyclicity and SHBG, reduces androgen levels, LH/FSH, and LH response to GnRH [143–145]. 

The relation between acupuncture, *β*-endorphin, and sympathetic activity seems to be sustained also in PCOS. Acupuncture treatment has been shown to reduce both high plasma *β*-endorphin and sympathetic nerve activity and increase low hand-skin temperature [30, 128, 146]. The involvement of sympathetic nervous system and an alteration in neurogenic control of the ovary have been implicated in the etiology of PCOS [111, 112, 147, 148]. One hypothetical mediator for that effect, combining an autonomic and ovarian effect, is nerve growth factor (NGF). Ovarian NGF production is increased in the follicular fluid in women with PCOS and in an estradiol-valerate- (EV-) induced rat PCOS model [92, 149, 150]. Over expressing NGF in the ovaries of a transgenic mice model results in ovarian hyperinnervation, arrested follicle growth, and increased ovarian steroidal responsiveness to gonadotropins [92]. Also ovarian adrenergic receptors have been shown to be increased in the EV-induced rat PCOS model further indicating involvement of sympathetic system in the regulation of ovarian function [151]. Electro-acupuncture has been shown to decrease high ovarian NGF [149, 150] and adrenergic receptors [152] in the EV-induced PCOS model and to decrease high mRNA expression of NGF and several markers of sympathetic activity in adipose in the DHT-induced PCOS model [153]. Together with microneurography measurements demonstrating a decrease of high MSNA in women with PCOS after 14 acupuncture treatments, this indicates an involvement of the sympathetic nervous system in both PCOS etiology and the mechanistic effect of acupuncture [30]. That naltrexone, a *μ*-receptor that antagonist induces ovulation and decreases LH concentration, indicates also a role of *β*-endorphin in PCOS [143–145]. That similar effects are also mediated by electroacupuncture [146, 154, 155] concurrent with a lowering of *β*-endorphin levels [146] implying the involvement of the opioid system in the underlying mechanism. This is also supported in our recent experimental study where electrical stimulation of the acupuncture needles affected the expression of opioid receptors *μ* and *κ* in rat hypothalamus, together with improved cyclicity and reduction of circulating testosterone levels [156]. 

## 5. Treatment of Reproductive Function in PCOS

Due to the uncertainty of the etiology of PCOS as well as the heterogeneity of the syndrome no cure can currently be offered. Women are instead treated in a symptom-oriented manner, often for long duration, with associated adverse effects.

Lifestyle modifications, including diet and exercise, are frequently recommended as a first-line treatment in the large population of overweight and obese PCOS women. It improves several of the key features of PCOS such as body composition, hyperandrogenism, and cardiometabolic profile, including insulin sensitivity and blood lipids, but also autonomic function and inflammatory pattern [157–166]. The effect may also include improved ovulatory function and pregnancy [158, 160, 163, 167, 168] alone or together with clomiphene citrate (CC) [162], and even a modest (5%) weight reduction has been reported to improve metabolic and reproductive functions [169]. 

For women who do not wish to conceive, combined oral contraception is frequently used for regulation of menstrual bleeding patterns and reduction of androgen levels [25]. However, use of oral contraceptives may be contraindicatory in women with PCOS suffering from obesity and/or insulin resistance or risk of cardiovascular disease [25]. 

Nonrandomized studies on acupuncture for women with PCOS as well as with undefined anovulation show improvements of menstruation pattern, LH/FSH ratio, estrogen, and testosterone [146, 154, 155]. The effect on ovarian function was first demonstrated in an RCT in which acupuncture with combined manual and low-frequency (2 Hz) electrical stimulation of needles during 30 minutes per treatment was superior to 16 weeks of exercise according to the Randeva protocol [170] and no intervention in improving menstrual frequency and lowering circulating levels of testosterone in PCOS women [166]. Needles were placed in abdominal and leg muscles in the same somatic segment as the innervation of the ovaries. The treatment period was 16 weeks and women received in total 14 treatments: twice a week during 2 weeks, once a week during 8 weeks, and thereafter every other week. Concurrently, Pastore et al. compared the effectiveness of true acupuncture and sham acupuncture using the Park device [171]. They received 12 treatments over 14 weeks and the needle placement and stimulation in the true acupuncture group followed an old protocol [172]. Both groups improved their menstrual frequency, and no difference between the groups was observed on ovulation rate during or after treatment, thus indicating that the sham device is not completely inert. In a more recent RCT, we further determined ovulation frequency in PCOS women, during a 3-month long acupuncture or attention control treatment period, by weekly progesterone measurement as well as menstrual bleeding registrations. In this study, women received acupuncture twice a week during the study period with similar needles placement as in the previous study, thus increased intensity in terms of more frequent treatment. The group who were allocated to acupuncture treatment had higher ovulation frequency compared with the attention control group [173], an effect that was augmented compared to the previous clinical studies on PCOS with fewer acupuncture treatments, indicating a dose-response effect [166, 171, 172]. In addition, the ovulation frequency in the attention control group [173] was comparable with ovulation frequency during CC stimulation [174], as well as the one in a previous acupuncture study on PCOS women [171]. Supporting animal data include improved ovarian morphology [153] and restored estrous cyclicity [47, 156] with increased progesterone levels [156] after acupuncture in a DHT-induced rat PCOS model [22]. Moreover, there were no significant differences in estrous cyclicity improvement between the two stimulation modalities: electrical and manual needle stimulations [156]. The parallel effect regarding ovulation in the rat and human studies strengthens the result by the translational nature. Proceeding to ovarian morphology in the recent RCT, no group differences were observed when counting antral follicles ≤9 mm or ovarian volume [173]. However, a tendency to reduced number of antral follicle and ovarian volume was observed within the acupuncture group [173]. This is also in line with previous animal studies where electroacupuncture improved ovarian morphology in DHT-induced PCOS rats [153]. In addition, we found reduced circulating levels of inhibin B after acupuncture, without changes in AMH [173] which was in line with a previous study [60]. This may either represent a decreased size of the follicular pool or mimic an improved follicular development and reduced levels of ovarian sex steroids [66–68, 71]. Moreover, delta changes of most circulating sex steroids (E_1_, E_1_-S, E_2_, T, free-T, DHT), sex steroid precursors (DHEA, DHEA-S) and glucuronidated androgen metabolites (ADT-G, AD3G, and AD17G), as determined by mass spectrometry differed between the intervention groups. Differences in E_1_-S, E_2_, DHEA, free-T, and ADT-G held for bonferroni correction. Acupuncture reduced levels of E_1_, E_1_-S, E_2_, DHEA, DHEA-S, 4-DIONE, T, and free T from baseline to end of treatment [173]. This is in line with, and extends, our previous RCT where several of these steroids were reduced [166]. Also here the clinical data is in line with the experimental data, where acupuncture with low-frequency electrical stimulation reduced levels of testosterone in DHT-induced PCOS rats [156]. The enhanced effect regarding both ovulation and sex steroids may be explained by increased number of treatments, more than 20 treatments, compared to the previous study comparing 14 treatments with acupuncture and exercise, as our animal studies infer dose-responsive effects [166, 173, 175, 176]. 

### 5.1. Gap in Knowledge

The present studies on menstrual and ovulation frequency have limited sample size and lack longitudinal followup for more than 16 weeks. It is also of importance to describe the most effective treatment regimen including maintenance treatment.

Treatment of first choice for ovulation induction in women with PCOS attempting to conceive is lifestyle interventions [177] and thereafter clomiphene citrate (CC) [178]. Ovarian hyperstimulation, multiple gestations, and spontaneous abortions (~20% instead of the normal 12–15%) belong to the adverse effects of CC stimulation, although rare [178, 179]. Approximate 20% of PCOS women are designated to be CC resistant [180, 181] and if CC fails, gonadotropin (FSH) therapy or laparoscopic ovarian surgery is considered as further treatment. The efficacy of gonadotropins is a 70% ovulation rate and a 20% pregnancy rate [178], but the treatment is costly and requires intensive ovarian monitoring [182]. Laparoscopic ovarian surgery (LOS), such as ovarian drilling by electrocautery or laser, has a pregnancy rate between 25–51% and a live birth between 24–44% [183]. However, these more invasive treatments are associated with side-effects such as adherence. Other used treatments for ovulation induction are considered in more detail in other review articles such as the ESHRE/ARSM sponsored PCOS consensus report from 2007 [178]. The earlier described acupuncture studies show improvement of menstrual and ovulatory function and may therefore also improve pregnancy rate in women with PCOS, either alone or in combination with traditional treatment [166, 171, 173].

### 5.2. Gap in Knowledge

Previous studies indicate that acupuncture induces ovulation and thus may lead to pregnancy in women with PCOS [166, 173]. However, none of these trials were designed to investigate pregnancy or live birth. This gap in knowledge is addressed in an ongoing, multicenter, head-to-head RCT (ClinicalTrials.gov: NCT01573858) in China. This trial tests the following hypotheses in anovulatory women with PCOS: (1) true acupuncture and CC are more likely to result in ovulation and live birth than control acupuncture and CC, (2) control acupuncture and CC are more likely to result in ovulation than true acupuncture and placebo CC, and (3) true acupuncture and placebo CC are more likely to result in live birth than control acupuncture and placebo CC.

### 5.3. Acupuncture on Neuroendocrine Function in PCOS

To elucidate possible explanations for the effect of acupuncture on ovulation in women with PCOS and DHT-induced PCOS rats studies measuring LH pulsatility and experimental studies exploring other neuroendocrine functions have been conducted with varying results [156, 173, 184].

In the rat, the highest density of GnRH neurons is located in the rostral medium septum (MS), diagonal band of Broca, and medial preoptic area (MPO) [185]. Most GnRH neurons send projections via mediobasal hypothalamus down to the median eminence where GnRH is released into pituitary portal blood [186, 187]. In DHT-induced PCOS rats, there seem to be more GnRH-immunoreactive (GnRH-ir) cells in the MPO and horizontal limb of the diagonal band (HDB) than in control rats [184]. Additionally, compared to controls, the PCOS rats displayed increased levels of hypothalamic functionally active androgen receptor (AR) and AR-ir cells in the MPO as determined by western blot and immunohistochemistry (IHC). This implies that androgens may have a regulatory effect on the GnRH neurons. Moreover, the increased levels of GnRH-ir cells and AR in hypothalamus were reduced after electroacupuncture treatment [184]. Estrogen receptor *β* has previously been shown to be co-localized with GnRH neurons, thereby indicating a direct action of estrogens on GnRH regulation [188]. It has also demonstrated a colocalization of AR and GnRH neurons that further strengthens the hypothesis of a direct androgenic regulatory control on GnRH neurons [184]. Hence, we have indications that both the GnRH abnormalities and the effect of electroacupuncture could be mediated via the AR in hypothalamus. Moreover, previous experiments have demonstrated that levels of circulating estradiol are not altered in the DHT-induced rat PCOS model, which further supports the conclusion that this is an androgenic effect [22]. 

Although the arcuate nucleus is considered to be the principal location of the GnRH pulse generator in the rat brain, the preoptic area regulates the GnRH secretion surge controlling the preovulatory LH surge [132, 189] making the findings in MPO even more interesting. Administration of androgens prenatally has previously been shown to increase LH pulsatility, without affecting pituitary responsiveness to GnRH, and to abolish the estrogen-induced LH surge, which is indicative of acceleration in the GnRH pulse generator. A contemporary reduction of estradiol-induced progesterone receptor (PR) expression in the preoptic area was then considered as evidence of how androgens could mediate this effect [190]. 4-day administration of androgens to adult rats produced similar results on the LH surge and PR expression but with the contrast of an instead decreased LH and possibly GnRH secretion, similar to the male response [191, 192] and in oppose to human PCOS. These results are indicative of a programming effect (defect) of androgens during prenatal development that is different from the direct effects of hyperandrogenemia in adult life. Since in the DHT-induced PCOS model, rats are continuous exposed to androgens from day 21 until adult life, it is possible that they also have reduced GnRH secretion, with abolished GnRH/LH surges [191, 192]. One might therefore speculate that restoration of the GnRH and LH surge by effects on the androgen receptor in the MPO could be at least part of the mechanism behind the improved estrous cyclicity after electroacupuncture. A succeeding study could not confirm the affected hypothalamic GnRH and AR expression; however, these measurements were made at mRNA level [156]. This could mean that the effect is related to posttranslational events rather than gene expression changes. However, no measurements of LH pulsatility have been performed in the DHT-induced rat PCOS model, either basally or after electroacupuncture, to confirm a physiological effect.

The hypothesis that the higher ovulation frequency by acupuncture in women with PCOS is due to restoration of gonadotropin secretion was tested in the recent RCT [173]. However, none of the LH pulsatility measures were affected by acupuncture treatment twice a week during 3 months. The major effect accompanying the higher ovulation frequency and reduced circulating inhibin B levels in the acupuncture group was the general decrease in circulating sex steroids, androgen precursors, and glucuronidated androgen metabolites after treatment, pointing towards an effect at ovarian and adrenal levels. However, it does not exclude involvement of central control mechanisms [173].

In an earlier RCT comparing sham and true acupuncture in PCOS women the LH : FSH ratio was decreased in both groups together with rather high ovulation frequencies [171]. The lack of effect on LH pulsatility in the latest RCT [173] may be due to the sampling timing in the menstrual cycle, which could be a confounding factor [44, 193, 194]. The majority of endpoint overnight blood sampling was performed during cycle day 8–10 (mid to late follicular phase). It is possible that an effect might have been seen if blood samplings were instead performed in early follicular phase [173]. 

## 6. Discussion

The viscous circle of PCOS features aggravating each other may be driven by androgens, insulin, or other factors but must be broken to improve the health status of women with PCOS. Although pharmacological treatment may be effective, they are also associated with negative side-effects [97]. This review addresses acupuncture as a potential treatment option for reproductive and endocrine disturbances in women with PCOS. Several clinical and animal experimental studies indicate that acupuncture is beneficial for ovulatory dysfunction in PCOS. This is also related to decreased levels of sex steroids and possibly inhibin B. Although the clinical data does not support changes in LH pulsatility/secretion pattern as a possible mediator for this effect [82], there is strong evidence of central components that probably involve both opioid and sympathetic activities, an effect that may be mediated via the androgen receptor. However, the causality of changes in ovulation and sex steroids is difficult to determine. The reduction of androgen levels by normalization of intrinsic ovarian aberrations may calm the exaggerated follicular growth, restore the follicle maturation processes, and hence lead to ovulation [12]. It could also be the other way around that improved ovulation, possibly caused by external factors, restoring follicular growth and/or aberrations and thereby reducing androgen levels due to the lower pool and/or activity of androgen producing cells [32].

Improved menstrual and ovulatory patterns are indeed beneficial, both for women not trying and women who are attempting to conceive. Further studies are needed to investigate whether acupuncture improves pregnancy and live birth rate in women with PCOS.

For the purpose of implementing acupuncture in the conventional treatment strategy, though, it is also of importance to compare it with the first-line pharmaceutical options. This is important for the support and sanction of acupuncture as a treatment method. To further investigate the underlying mechanisms of the effect of acupuncture may also be supportive in the search for other possible alternative treatments, including pharmacological. 

## Figures and Tables

**Table 1:** Diagnostic criteria of PCOS.

Diagnostic criteria	NIH 1990	Rotterdam 2003	AES 2006
	Require	At least two out of	Require
Hyperandrogenism (HA)^A^	*√*	*√*	*√*
Ovulatory dysfunction (OD)^B^	*√*	*√*	and/or *√*
PCO morphology (PCO)^C^		*√*	and/or *√*

Possible phenotypes	(1) HA + OD	(1) HA + OD + PCO (2) HA + OD (3) HA + PCO (4) PCO + OD	(1) HA + OD + PCO (2) HA + OD (3) HA + PCO

^A^Clinical or biochemical signs of hyperandrogenism.

^B^Oligomennorrhea, amenorrhea, oligoovulation, or anovulation.

^C^≥12 follicles of 2–9 mm and/or enlarged ovarian volume of  ≥10 mL in one or both ovaries.
